# An AUG Codon Conserved for Protein Function Rather than Translational Initiation: The Story of the Protein sElk1

**DOI:** 10.1371/journal.pone.0102890

**Published:** 2014-07-18

**Authors:** Noemie Legrand, Tanguy Araud, Beatrice Conne, Odin Kuijpers, Pascale Jaquier-Gubler, Joseph Curran

**Affiliations:** 1 Department of Microbiology and Molecular Medicine, University of Geneva Medical School, Geneva, Switzerland; 2 Department of Genetic Medicine and Development, University of Geneva Medical School, Geneva, Switzerland; 3 Institute of Genetics and Genomics in Geneva (iGE3), University of Geneva, Geneva, Switzerland; University of British Columbia, Canada

## Abstract

Elk1 belongs to the ternary complex (TCF) subfamily of the ETS-domain transcription factors. Several studies have implicated an important function for Elk1 in the CNS including synaptic plasticity and cell differentiation. Whilst studying *ELK1* gene expression in rat brain a 54 aa N-terminally truncated isoform lacking the DBD was observed on immunoblots. A similar protein was also detected in NGF differentiated PC12 cells. It was proposed that this protein, referred to as sElk1, arose due to a *de-novo* initiation event at the second AUG codon on the Elk1 ORF. Transient over-expression of sElk1 potentiated neurite growth in the PC12 model and induced differentiation in the absence of NGF, leading to the proposition that it may have a specific function in the CNS. Here we report on the translational expression from the mouse and rat transcript and compare it with our earlier published work on human. Results demonstrate that the previously observed sElk1 protein is a non-specific band arising from the antibody employed. The tight conservation of the internal AUG reported to drive sElk1 expression is in fact coupled to Elk1 protein function, a result consistent with the Elk1-SRE crystal structure. It is also supported by the observed conservation of this methionine in the DBD of all ETS transcription factors independent of the N- or C-terminal positioning of this domain. Reporter assays demonstrate that elements both within the 5′UTR and downstream of the AUG^Elk1^ serve to limit 40S access to the AUG^sElk1^ codon.

## Introduction

Elk1 belongs to the ternary complex (TCF) subfamily of the ETS-domain transcription factors. In cultured cells, it functions as a transcriptional activator via its association with serum response factor (SRF) in a ternary complex on the serum response element (SRE) of many immediate early genes (IEGs: e.g. c-fos. egr1, egr2 and pip92). The TCFs are major nuclear targets for MAPKs (mitogen-activated protein kinases) of the extracellular-signal regulated kinase (ERK) subfamily, and the closely related SAPK/JNK and p38MAPK stress-activated protein kinases. They therefore appear to act as an integration point for both growth and stress signals [1]. The Elk1 protein is 428 amino acid (aa) long (52 kDa) and its N-terminal 86 aa harbours the ETS DNA binding domain (DBD) that recognises an 11 base-pair sequence containing a central 5′-CAGGA motif that forms part of the SRE.

In the CNS, Elk1 is activated by ERKs and these kinases respond to neurotrophins and neurotransmitters. ERKs are abundant in this tissue and play important roles in neuronal survival, synaptic plasticity, long-term behavioural changes and memory learning. These functions seem to be associated with IEG induction (in particular *c-fos*), and this in-turn suggests a central role for Elk1 in these processes [2]–[5]. In post-mitotic neurones Elk1 is detected in both the nucleus and cytoplasm, including neuritic extensions, whereas in transiently transfected Hela cells it is exclusively nuclear. Whilst studying the localization of Elk1 in the brain, a novel isoform sElk1 (short Elk1) was reported [6]. It was proposed that it was generated by a de-novo initiation event at the second AUG in the Elk1 open reading frame (ORF). This 45 kDa protein was expressed uniquely in brain tissue and in nerve growth factor (NGF) differentiated PC12 cells. Intriguingly, its localization was exclusively nuclear. The 54 aa N-terminal deletion in sElk1 removes most of the DBD, explaining why it was severely compromised in its ability to activate SRE genes. Curiously, in NGF treated PC12 cells sElk1 expression correlated with the redistribution of Elk1 into the cytoplasm. In addition, over-expression of sElk1 from a cDNA clone induced PC12 differentiation in the absence of NGF [6]. It was proposed that both Elk1 and sElk1 exercise important functions in the brain possibly coupled to the regulation of neurone differentiation.

Our lab has exploited the *ELK1* mRNA as a model system to examine translational regulation mediated via the 5′UTR. We earlier characterised two alternatively spliced 5′UTRs of the human gene (referred to as 5′UTR^L^ and 5′UTR^S^) whose relative abundance showed tissue specificity. Both contain uAUGs, uORFs (open reading frames) and RNA structure, however, we observed no IRES activity [7]. The human *ELK1* gene contains seven exons (E1 to E7). The protein sequence is encoded within E3 to E7. E1, E2, and the first 34 nts of E3 constitute the 5′UTR of the long variant (5′UTR^L^). The E2 is absent in the short (5′UTR^S^). E1 is highly GC rich (>75%) and within the mature mRNA folds into a number of stable stem-loops (SL) with Δ*G*° approaching −40 kcal/mol [7]. Ribosome access to the human sElk1 (AUG^sElk1^) requires the by-passing of three out-of-frame AUGs positioned downstream of the AUG of Elk1 (AUG^Elk1^), two of which have good Kozak sequences (referred to hereafter as internal AUGs a/b/c: iAUG^a/b/c^). In our earlier studies, we proposed that this could be achieved by delayed reinitiation events mediated by the small uORF2 (2 codons) positioned only 14 nts upstream of the AUG^Elk1^. This uORF2 is conserved in all mammalian genes. Using reporter systems that monitored initiation events at the human AUG^Elk1^, AUG^sElk1^ and iAUG^a/b/c^, we demonstrated that: 1. Initiation events at the AUG^Elk1^ arose by a combination of leaky scanning and reinitiation. 2. In normal growing HEK293T cells the major translation products arose due to initiation at the iAUG^a/b/c^. All were the result of delayed reinitiation mediated by uORF2. Under these conditions no initiation events were detected at the AUG^sELK1^. 3. Lowering the eIF2.GTP.tRNA^Met^ ternary complex (TC) levels dramatically reduced the overall readout in the reporter assays but pushed delayed reinitiation towards the 3′. This allowed us to detect weak initiation events in HEK293T cells at the AUG^sElk1^ [8].

Taken together, these observations lead us to propose that a similar mechanism would be functioning in rodent neuronal tissue. We have now tested this in the mouse N2a neuronal cell line in which we appeared to observe high and constitutive expression of a sElk1 protein as detected by immunoblotting employing the same antibody used in the original report [9]. This has been combined with an analysis of the 5′ end sequences of the mouse and rat genes. Our results indicate that the sElk1 protein band detected in immunoblotting is non-specific and that sElk1 expression in both the murine N2a cells and differentiated rat PC12 cells is below the level of detection independent of TC levels. Indeed, initiation events at the AUG^sElk1^ are even more repressed in the rodent background due to the presence of an additional iAUG (iAUG^d^ absent in human) positioned between iAUG^c^ and AUG^sElk1^. We demonstrate that the AUG codon for sElk1 is actually conserved because of its function within the Elk1 ETS DNA binding domain, a result consistent with the Elk1-SRE crystal structure [10]. Our results indicate that within the mammalian *ELK1* gene an elaborate mechanism has been put in place to limit 40S ribosomal access to this internal AUG codon in the delayed reinitiation mode.

## Materials and Methods

### Cell culture and transfection

HEK293T (ATTC, CRL-11268) and N2a cells (ATCC, CCL-131) were cultured in Dulbecco's modified Eagle's medium (Sigma) supplemented with 10% foetal calf serum (FCS) (Brunschwig), 1% penicillin/streptomycin (Gibco), in a humidified atmosphere containing 5% CO_2_. PC12 cells (ATCC, CRL-1721) were grown on collagen coated plates in RPMI-1640 medium supplemented with 10% horse serum (Sigma), 5% FCS and 1% penicillin/streptomycin. For differentiation, neuronal growth factor (NGF, Alomone labs) was added at 50 ng/mL under the serum conditions indicated in the text. Neurite outgrowth was visible three days later. N2a cells were transfected using Lipofectamine 2000 (Life Technologies). Transfections were performed in normal growth medium when the cells were no more than 50% confluent. Four hours later the medium was replaced with fresh growth medium except in those experiments in which the serum concentration was altered. Cytoplasmic extracts were prepared by solubilizing the monolayer in 150 mM NaCl, 50 mM Tris–HCl pH 7.4, 10 mM EDTA, 0.6% NP-40, and the complete mini protease inhibitor cocktail (Roche). Nuclei were removed by pelleting at 20,000 g for 5 min. Protein concentrations were determined by Bradford assay (Cytoskeleton, USA). Transfections were generally carried out at least in duplicate and repeated at least twice.

### DNA constructions

Unless indicated all clones were in a pcDNA3 backbone. Cloning of the murine Elk1 was performed by RT-PCR using the primer set

5′ATCGGTACCACGTGAGCGGTGGGGAAACG 3′

5′CCGCTCGAGTGTTTCTCCTTGAGATGGCT 3′.

It was cloned KpnI/XhoI into pcDNA3 and lacks most of the 3′UTR. The murine LP^Next^ fusion clones were generated by PCR using the following primer sets:

5′ATCGGTACCACGTGAGCGGTGGGGAAACG 3′

5′GAGACGTGCTGAGTCCATGGTGGTCTTGTTCTTGCG 3′

5′GAGACGTGCTGAGTCCATGGTGGTCTTGTTCTTGCG 3′

### Western blot

Thirty µg of protein was resolved on a polyacrylamide-SDS gel and electro-transferred to PVDF. Antibodies used in this study were, anti-ELK1 (Santa Cruz #sc-355: referred to as Ab^SC^), anti-ELK1 (Abcam #ab131465: referred to as Ab^Ab^), anti-HA (clone 16B12, Covance), anti-eIF2α (Invitrogen #44728G), anti-phospho (Ser51) eIF2α (Cell Signaling #9721), anti-actin (clone C4, Millipore) and goat anti-mouse or rabbit HRP secondary antibody (Bio-Rad). Blots were developed using the Super Signal Substrate (Thermo Scientific) and quantified using the Quantity One software package (Bio-Rad).

### siRNA knockdown

The siRNAs against human (sc-35290) and mouse (sc-35291) Elk1 were purchased from Santa Cruz Biotech. Transfections were performed using Lipofectamine2000 reagent according to the suppliers protocol. Cells were harvested at 24 hrs post-transfection and processed for immunoblotting.

### ExonII

RNA was prepared using the TriZol reagent (Invitrogen). It was treated with DNase (Ambion DNA-free) and quality checked on an Agilent 2100 bioanalyser. The presence of an exonII was determined by RT–PCR using oligos positioned in exonI and III using the One Step RT–PCR kit (Qiagen) according to the manufacturer's instructions. For semi-quantitative RT–PCR the number of amplification cycles was first determined for each primer set, and corresponded to the exponential phase of the different products [7]. The same primer set was used for murine and rat (5′ACCGCCGCGCCACAC3′/5′ACCGCCGCGCCACAC3′).

### CHIP analysis

Briefly, 10^7^ cells were cross-linked with 1% formaldehyde for 10 minutes at room temperature. The reaction was quenched with 0.125 M glycine for 10 minutes. Cells were resuspended in 1 mL of lysis buffer (5 mM PIPES pH 8.0, 85 mM KCl, 0.5% IGEPAL, 1X protease cocktail inhibitor (Roche) and incubated 30 min at 4°C with agitation. Nuclei were pelleted and resuspended in 300 µL of nuclei lysis buffer (50 mM Tris-HCl pH 8.1, 10 mM EDTA, 1% SDS, 1X protease cocktail inhibitor). The soluble chromatin with a size range of 0.5 kbps to 0.9 kbps was prepared by sonication using a Bioruptor (Diagenode) at the following settings: 5 cycles of sonication (10 seconds on /40 seconds off). Supernatant was pre-cleared 1 h at 4°C with 25 µL of a 50% gel slurry of protein A/G–agarose beads saturated with salmon sperm DNA and bovine serum albumin. Supernatant was recovered, diluted 10 times in IP dilution buffer (0.01% SDS, 0.5% Triton X-100, 2 mM EDTA, 16.7 mM Tris-HCl pH 8.1, 100 mM NaCl, 1X protease inhibitor cocktail) and 10% was used as input. Diluted extracts were incubated overnight with 1 µL Ab^SC^ and the immune complexes recovered on 15 µL of protein A/G-agarose beads (Millipore). Chromatin was eluted twice with 60 µL of freshly prepared elution buffer (100 mM NaHCO_3_, 1% SDS). The crosslinking was reversed by an overnight incubation at 67°C in the presence of 0.3 M NaCl and 10 µg of RNase A [10 µg/µL]. Samples were then digested with 30 µg of proteinase K [10 µg/µl] in the presence of 40 mM Tris-HCL pH 6.5 and 10 mM EDTA at 45°C for 1 h. DNA was purified using phenol-chloroform and analysed by real-time-PCR. The c-fos specific primers employed were as follows: Forward: 5′ GAGCAGTTCCCGTCAATCC 3′ Reverse: 5′ GCATTTCGCAGTTCCTGTCT

## Results

### sElk1 expression in rodent cells

**Figure 1:**
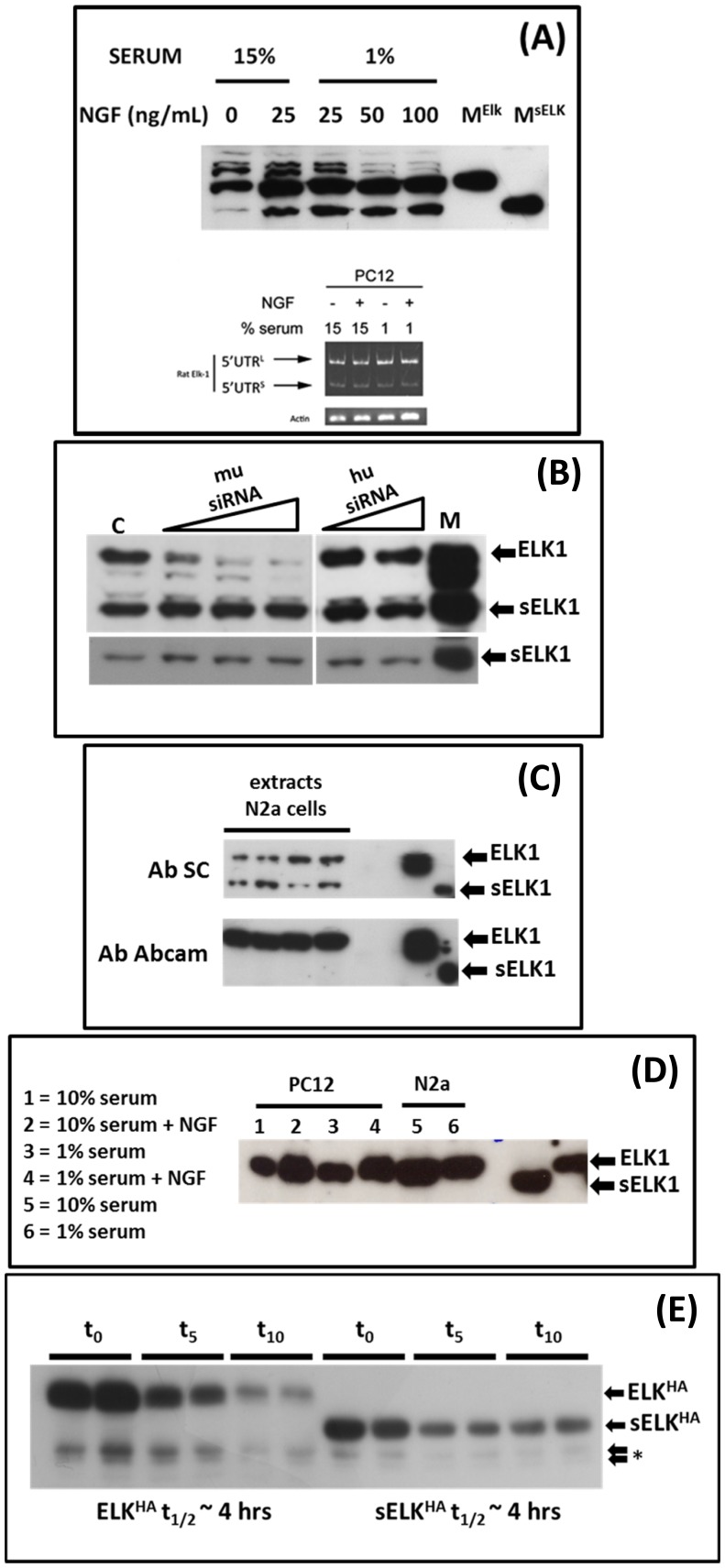
Elk1 expression in rodent cells. (A). Upper Panel: PC12 cells were treated with increasing doses of NGF at low or high serum concentrations. Cell extracts were prepared 5 days after NGF treatment and analysed by immunoblotting with the anti-Elk1 Ab^SC^. M^Elk^ and M^sElk^ are markers for these proteins generated by transiently over-expressing cDNA clones of each in HEK293T cells. Lower panel: RT-PCR was performed on total RNA extracted from PC12 cells cultured under the conditions indicated. Primers were positioned within the exon I and exon III. Amplicons corresponding to the 5′UTR^L^ and 5′UTR^S^ are indicated as is an actin control. (B). N2a cells were transfected with increasing doses of either a murine (0.1 nmoles, 0.2 nmoles and 0.3 nmoles/well) or human siRNA (0.2 nmoles and 0.3 nmoles/well) directed against the *ELK1* transcript. Cells were harvested 24 hrs post-transfection and analysed by immunoblotting using the Ab^SC^. The lane C is the non-transfected control, the lane M contains protein markers for Elk1 and sElk1. The lower panel is a shorter exposure of the sElk1 band. (C). Multiple independent N2a cells extracts were immunoblotted and probed with the Ab^SC^. The blot was stripped and re-probed with the Ab^Ab^. Markers for Elk1 and sElk1 are indicated. (D). Cell extracts prepared from PC12 cells grown under different serum concentrations +/− NGF and N2a cells were analysed by immunoblotting using the Ab^Ab^. Markers for Elk1 and sElk1 are indicated. (E). cDNA clones expressing Elk1^HA^ and sElk1^HA^ were transiently expressed in N2a cells. At 16 hrs post-transfection cells were treated with cycloheximide (10 µg/mL) and harvested at the times indicated (in hrs). Proteins were analysed by immunoblotting with the HA Ab. The bands were quantitated and the calculated half-life for each is indicated (see Figure S1). The * indicates HA-tagged products migrating faster than sElk1^HA^.

Using an Elk1 C-terminal antibody (Santa Cruz Biotechnology: referred to hereafter as Ab^SC^) a 45 kDa sElk1 protein was reported to be expressed in both the rat cerebral cortex and in differentiated rat PC12 cells [6]. The proposed AUG initiation codon corresponded to methionine 55 within the ETS binding domain of Elk1. Using the same antibody we also observed a band in the PC12 model that co-migrated with a transiently expressed cDNA marker for sElk1, and noted that its expression was triggered by neuronal growth factor (NGF) treatment independent of the serum conditions (Figure 1A). We sought to confirm the origin of the sElk1 protein using siRNA knockdown. However, transfection into PC12 cells during differentiation proved technically difficult. We therefore sought a more robust model system in which the sElk1 band could be detected under normal growth conditions. This was achieved with the murine N2a neuroblastoma cell line (Figure 1B). These cells have very high endogenous P-eIF2α levels compared to HEK293T cells (the human cell line in which our earlier studies on the human transcripts were performed and in which one does not normally observe sElk1 expression) and it seemed reasonable to assume, based upon our published model from the human gene, that this could explain the delayed reinitiation event required for ribosomal access to the AUG^sElk1^ [8]. However, whereas we could knock-down the band in N2a cells that co-migrated with our Elk1 marker using a murine but not a human siRNA, this same siRNA had no impact on the band co-migrating with the sElk1 marker (Figure 1B). We therefore employed a second commercial anti-Elk1 C-terminal Ab (Ab^Ab^). This Ab detected both transiently expressed Elk1/sElk1 markers. However, in N2a cells it only detected the band corresponding to the endogenous Elk1 (Figure 1C). Likewise, in PC12 cells under various serum conditions and +/− NGF only the Elk1 protein was observed (Figure 1D: compare with Figure 1A).We C-terminally HA-tagged the murine Elk1 (Elk1^HA^) and used it to generate a sElk1^HA^ construct (by PCR deletion cloning). Both were them transiently expressed in N2a cells. Time course studies indicated that both Elk1/sElk1 had similar half lives in this cell background (Figure 1E) with values in line with those reported by others for Elk1 [11]. Curiously, the cDNA clone expressing the full-length Elk1^HA^ protein failed to express any band co-migrating with sElk1^HA^ despite the presence of the small uORF2 (the source of reinitiating ribosomes) and despite evidence of initiation events downstream of the AUG^sElk1^ (Figure 1E indicated as a *). The analysis also precluded any product-precursor relationship.

### mRNA characterization

**Figure 2:**
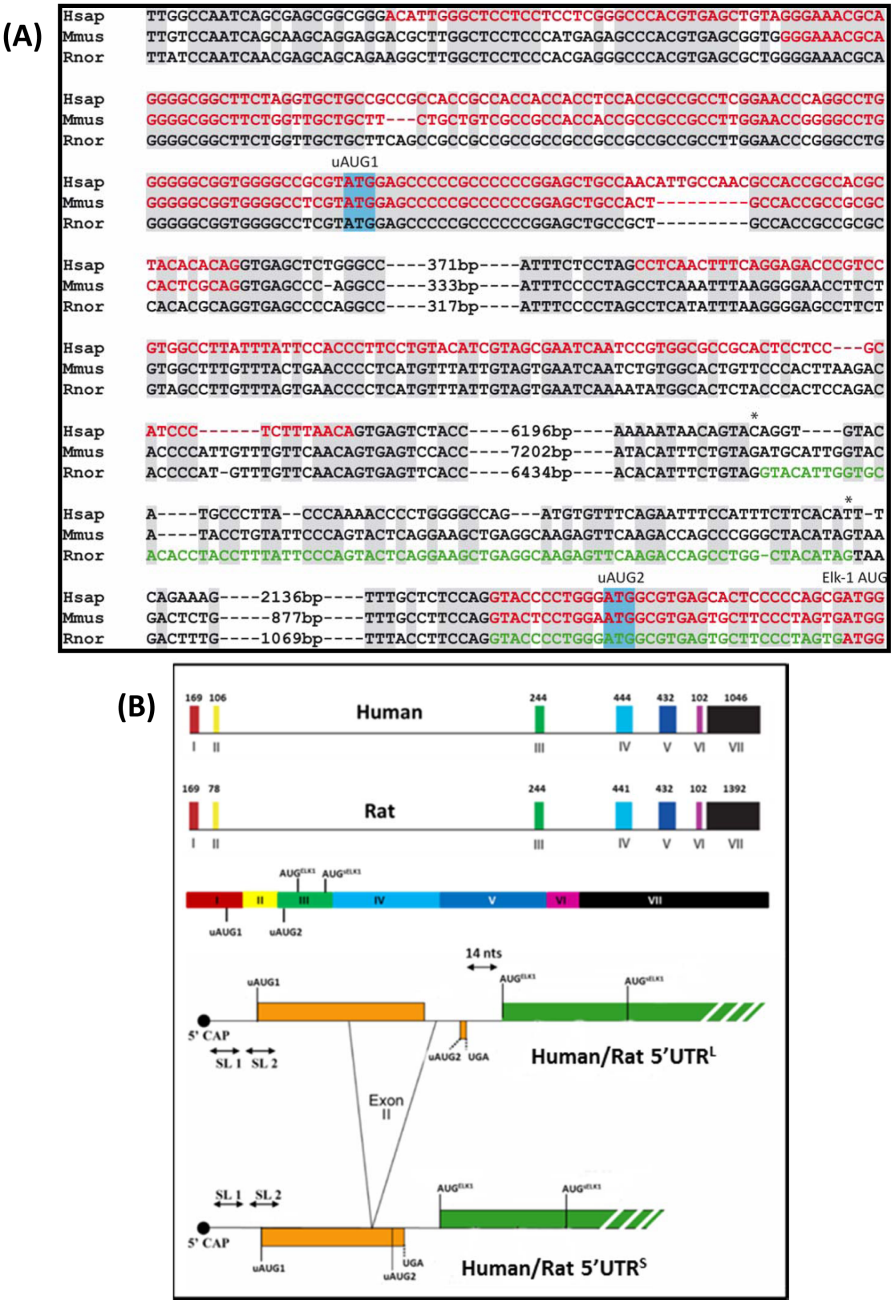
Characterisation of the *ELK1* gene. (A). Alignment of the genomic sequences at the 5′ extremity of the *ELK1* gene between Human (Hsap), Mouse (Mmus) and Rat (Rnor). Conserved bases are highlighted in grey. The sequence referred to as exons in the Ensembl Database are in red and the sequence of the exon II/III that we cloned and sequenced from PC12 cells is presented in green. The positions of the uAUG1, uAUG2 (boxed in blue) and AUG^Elk1^ are indicated. The * indicates the splice boundaries of the rat exon II. (B). Upper portion: Exonic organisation of the human and rat *ELK1* gene with the sizes of each exon (bps) and the positioning of key AUG codons indicated. Lower portion: A schematic representation that highlight's the conservation in the organisation of the 5′UTR^L^ and 5′UTR^S^ *ELK1* transcripts in both human and rat. SL1 and SL2 refer to the stable stem-loop structures around uAUG1 and 14 nts is the spacing between uORF2 and the AUG^Elk1^.

**Figure 3:**
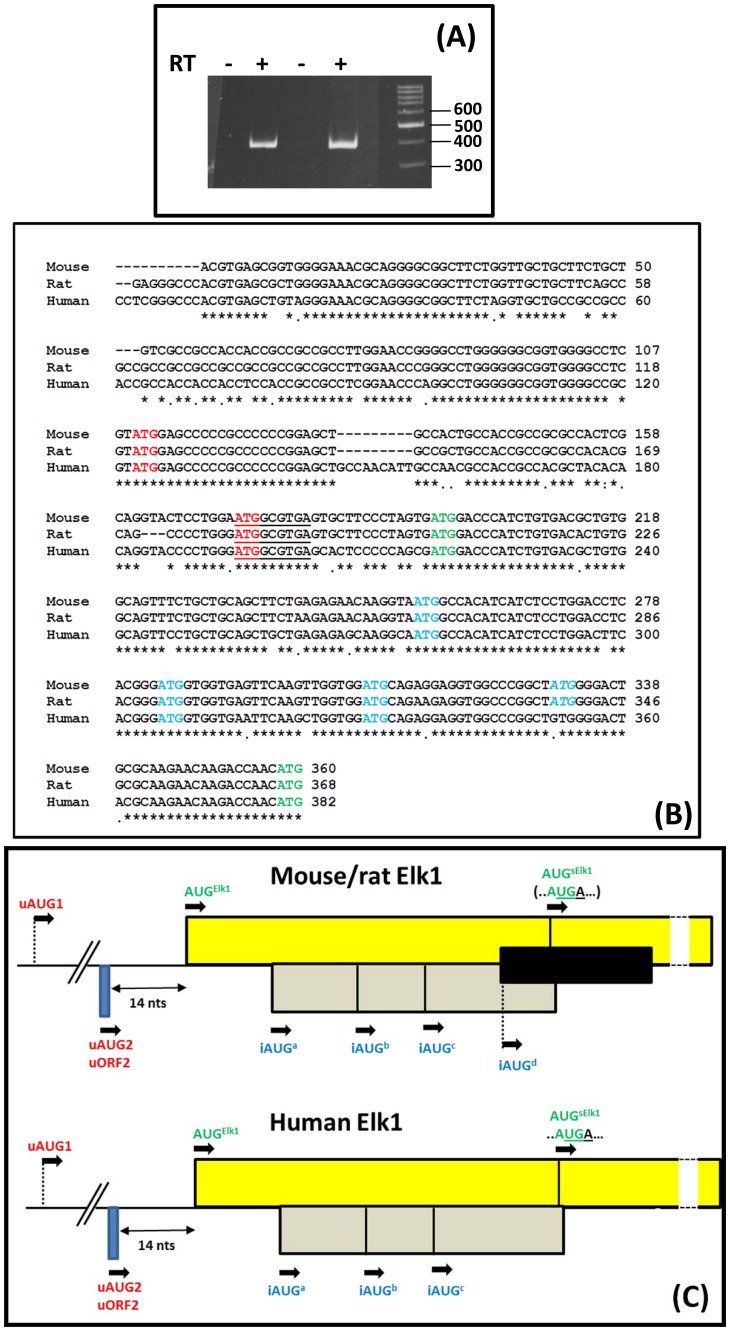
Analysis of the *ELK1* transcript in N2a cells. (A). RT-PCR amplification from total RNA isolated from N2a cells using primer sets in regions corresponding to the rat exon I and exon III. The experiment has been performed on duplicate samples and an RT minus control is included. (B). Alignment of the mouse, rat and human cDNA sequences up to the AUG^sElk1^ for the 5′UTR^S^. The uAUGs are indicated in red, the iAUGs in blue and Elk1/sElk1 AUGs in green. The small uORF2 is underlined. (C). A schematic representation indicating the positioning of the iAUGs in both mouse/rat and human transcripts. The Elk1 ORF is indicated above the line and the iAUG^a/b/c^ ORF below the line. The black rectangle in the mouse/rat Elk1 indicates the third ORF that can be expressed from the iAUG^d^. The colour coding for the AUGs corresponds to that depicted in B. The sequence below the AUG^sElk1^ demonstrates how this codon overlaps the UGA stop codon for the iAUG^a/b/c^ ORF. The small highly conserved uORF2 derived from uAUG2 is also depicted. It is the major source of reinitiating ribosomes that scan downstream of the AUG^Elk1^ [8].

RT-PCR analysis of RNA isolated from PC12 cells, using a primer set in the regions corresponding to the exon I and III, revealed the presence of the two alternatively spliced variants as observed previously in human cells (Figure 1A, lower panel) [7], [8]. The relative abundance of the two variants did not appear to change in response to NGF. We isolated, cloned and sequenced the larger RT-PCR product corresponding to the PC12 5′UTR^L^. This revealed that the rat exon II was different from that observed in human. Genomic sequence alignment of human, rat and mouse showed considerable conservation of the human exon II region in both rodent species, including the splice donor/accepter sites, however, the splicing signals around the rat exon II region, although largely conserved in mouse (although the 5′ splice donor site was sub-optimal: ..AG/AUG.. and not ..AG/GUA…), were lost in human (Figure 2A). Despite the change in the rat exon II, the overall organisation of the human and rat 5′UTR^L^ and 5′UTR^S^ was conserved, with the former retaining the two uORFs (the first is now shorter in rat, namely, 32 codons versus 54 codons in human) (Figure 2B). RT-PCR analysis of RNA isolated from N2a cells demonstrated the presence of only a single 5′UTR variant (using the same primer set employed for the rat) (Figure 3A) and sequencing confirmed that it corresponded to the 5′UTR^S^ observed in human and rat i.e. we were unable to detect a spliced variant carrying an exon II (Figure 3B). Alignment also highlighted the presence of a fourth internal AUG (iAUG^d^) in the rodent but not the human gene positioned just 25 nts upstream of the AUG^sElk1^ (Figure 3B/C). This iAUG^d^, which is in the third ORF, has the potential to function as a further translational repressor for sElk1 expression.

**Figure 4:**
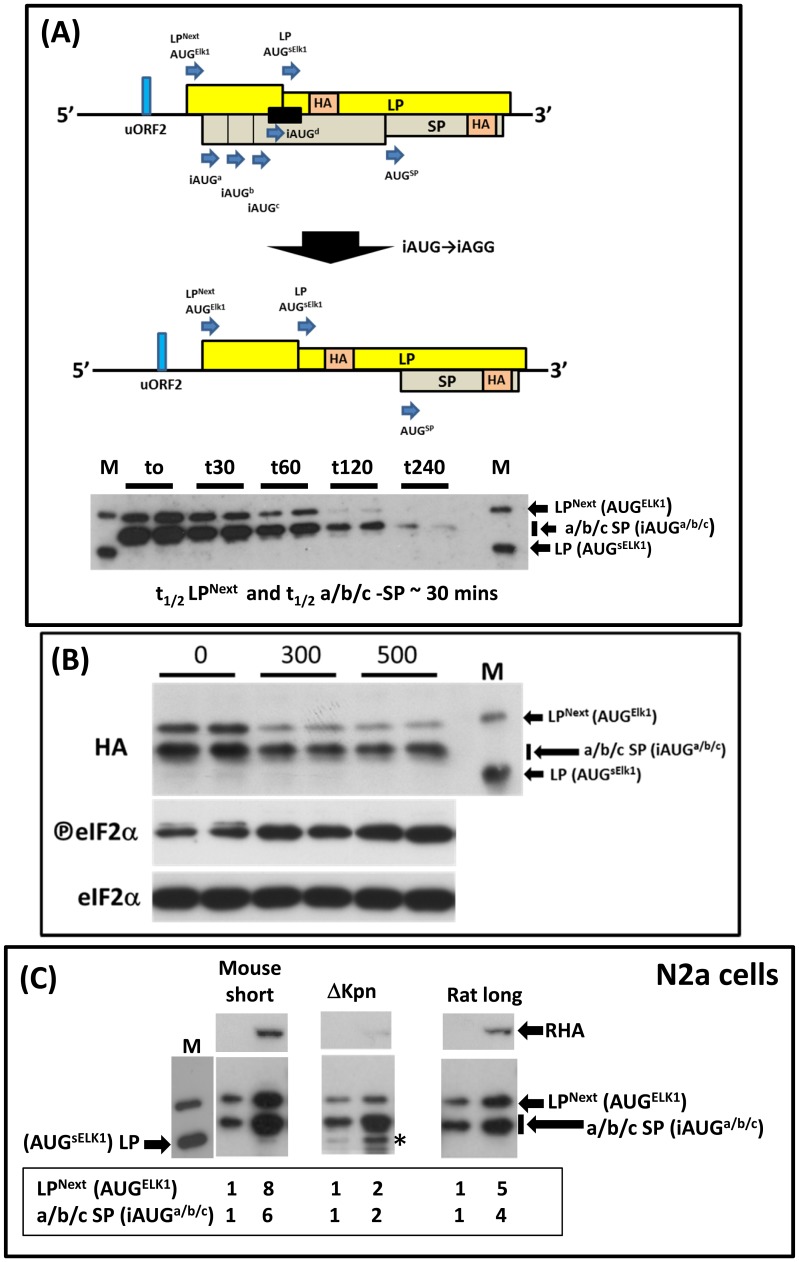
Initiation events at the AUG^sElk1^ in N2a cells: (A). Schematic representation of the LP/SP reporter used to monitor initiation events at the AUG^ELK1^ (LP^Next^), AUG^sElk1^ (LP) and iAUG^a/b/c^ (a/b/c SP). The position of the HA epitope tag in both the LP and SP fusion products is indicated as is the ORF derived from the iAUG^d^ found in the rodent *ELK1* gene (black rectangle). Mutation of the iAUGs to AGG dramatically alters the coding capacity of the LP/SP reporter. The lower panel depicts an immunoblot developed with the anti-HA Ab. N2a cells were transfected with the murine LP^Next^ clone. At 16 hrs post-transfection the cells were incubated with cycloheximide (10 µg/mL) and harvested at the time points (in minutes) indicated (see Figure S1). M refers to an LP^Next^ construct in which the iAUG codons have been mutated to AGG. It provides a size marker for initiation events at the AUG^sElk1^ (LP). The estimated half-life for the LP^Next^ and a/b/c-SP protein products estimated from the immunoblot is indicated below the gel. (B). N2a cells were transfected with the murine 5′UTR-LP^Next^ construct in the presence of increasing doses of thapsigargin (nM). Cells were harvested 20 hrs post-transfection and immunoblots performed with the indicated Abs. Transfections were performed in duplicate. M is a size marker for the LP^Next^ (AUG^Elk1^) and LP (AUG^sElk1^) protein products (see A). (C). LP^Next^ reporters carrying the murine 5′UTR (indicated as short), the murine ΔKpn (lacking exonI) and the rat 5′UTR^L^ (long) were transiently expressed in N2a cells in the absence or presence of a pcDNA3 plasmid expressing HA-tagged RHA. Cells were harvested 24 hrs post-transfection. Immunoblots were developed using the anti-HA Ab and the major bands, namely LP^Next^ and a/b/c-SP, were quantitated. These relative values are indicated in the panel below each blot. The * indicates the position of the AUG^sElk1^ initiation product LP. M provides size markers for the LP and LPNext products (see Figure 4A). Note the exposure time for both ΔKpn blot is much shorter as evidenced by the weakness of the RHA band.

The absence of initiation events at the AUG^sElk1^ was also reproduced using the LP/SP reporter vector which permits monitoring of multiple independent initiation events from overlapping ORFs [8], [12], [13]. Sequences upstream of the murine AUG^sElk1^ (Met55) were introduced 5′ of the LP AUG codon such that the Elk1 and LP ORFs were fused (Figure 4A). Because this fusion occurred via an NcoI site engineered around the AUG^sElk1^(.AAC**ATG** AA.→.ACC**ATG** GA.) the UGA stop codon for the iAUG^a/b/c^ ORF (underlined) was changed to UGG, a change that now fused this ORF to that of SP. As a consequence, this reporter can monitor initiation events at the AUG^Elk1^ (generating an LP^Next^ product), the AUG^sElk1^ (LP product) and iAUG^a/b/c^ (the a/b/c-SP products) by immunoblotting using the anti-HA Ab (Figure 4A). Time course analysis in transfected N2a cells revealed that the LP^Next^ and a/b/c-SP protein products had similar t_1/2_ (∼30 mins); as a consequence, the steady-state levels observed in immunoblotting reflected the rates of synthesis (Figure 4A, lower panel). As with the transiently over-expressed murine Elk1^HA^ cDNA clone we observed no protein product arising from the AUG^sElk1^ (LP), and this did not change even when phospho-eIF2α levels were increased by thapsigargin treatment of the cells (Figure 4B). Indeed, to observe a product from the AUG^sElk1^ it was necessary to mutate the iAUGs (iAUG→AGG: lane M in Figure 4A/B), a result that highlights there role in repression. As reported for the human gene, the major initiation events occur at these iAUGs [8].

Our earlier studies on the human gene had demonstrated initiation events at the AUG^sElk1^ upon deletion of exonsI and II, an event we attributed to the removal of repressional elements (e.g. the RNA structural elements within exonI) upstream of the uORF2 [7], [8]. A similar deletion (referred to as ΔKpn) generated in the murine LP/SP reporter also permitted weak but detectable initiation events at the AUG^sElk1^ (Figure 4C). We tested the contribution of simple de-repression by replacing the deletion mutant with the co-transfection of RNA helicase A (RHA). This DExH helicase (also called DHx9) has been reported to selectively stimulate translational expression from transcripts carrying structured 5′UTRs [14]. Additionally, since sElk1 was reported in the rat PC12 cell line that expresses a 5′UTR^L^ carrying a distinct exonII we also tested this variant in the assay (Figure 4C). Despite a significant increase in the reporter signals upon co-expression of RHA, we observed little evidence for initiation events at the AUG^sELK1^ with either the murine 5′UTR or rat 5′UTR^L^ constructs. However, all protein products in the ΔKpn background, including LP, were increased in the presence of RHA. It should be noted that the exposure time of the ΔKpn blot is shorter (note the intensity of the RHA band) and has been selected so that the LP^Next^ and a/b/c-SP products have similar intensities for all three constructs tested. This indicates that sequence elements within exon I appear to be modulating the behaviour of initiation events downstream of uORF2, further restricting 40S access to the AUG^sElk1^. This result is in-line with our earlier observations for the human transcript [8].

The absence of significant initiation events at the AUG^sElk1^ in transient expression studies using both tagged Elk1 cDNAs and reporter vectors is therefore consistent with the immunoblots performed using the anti-Elk1 Ab^Ab^ and the siRNA knockdown.

### Mutation of the methionine^55^ impairs Elk1 binding to the c-fos promoter

**Figure 5:**
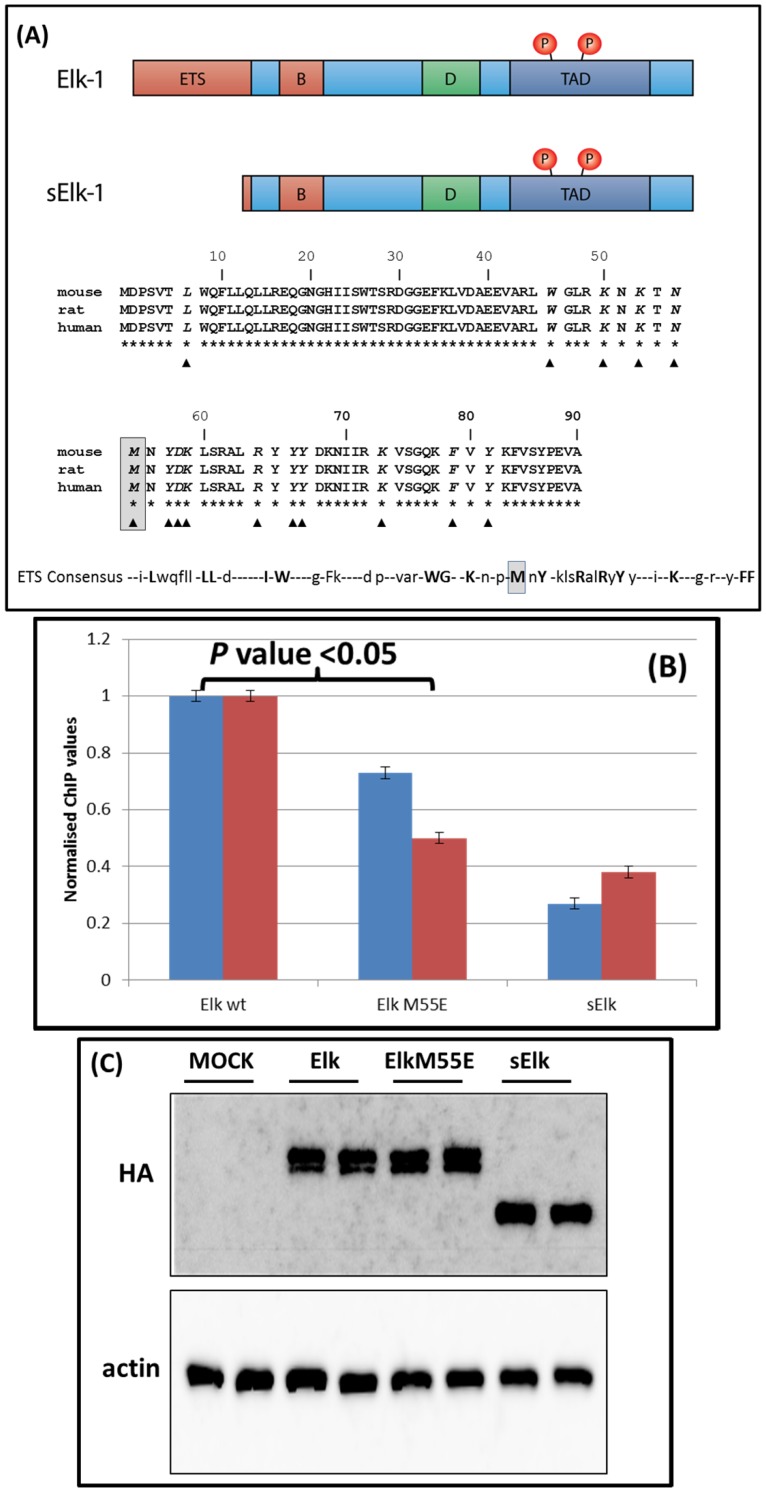
Function of the methionine 55. (A). Organisation of the Elk1 protein. Upper panel: Protein functional domains (ETS  =  DNA binding, B =  SRF interaction, D =  MAPK docking site, TAD  =  transactivation domain). Middle panel: Alignment of the Elk1 ETS domain from mouse, rat and human. The residues that make conserved DNA backbone and base contacts and are common to both Elk1 and SAP1 are indicated by the inverted triangles. The methionine corresponding to the AUG^sElk1^ is boxed in grey [10]. Lower panel: A consensus ETS domain based upon the alignment of 12 family members with DBDs both N- and C-terminal. Tightly conserved amino acids are indicated in bold capitol. The methionine corresponding to the AUG^sELK1^ is boxed in grey (adapted from http://content.lib.utah.edu/utils/getfile/collection/etd1/id/1528/filename/1687.pdf) (B). Chip analysis at the c-fos promoter. HEK293T cells were transfected with plasmids expressing ELK1^HA^, sElk1^HA^ or the Elk1^HA^ M55S mutant. ChIP analysis coupled to qPCR was performed as outlined in the supplemental section. The assay was performed twice (the duplicate columns) and analysed by qPCR. The ΔCt values (relative to input) were then normalised relative to Elk1^HA^ which was given a value of 1 in each experiment. (C). Immunoblot confirming expression from the transfected plasmids. Mock indicates non-transfected cells.

The Elk1 protein is a member of the ETS transcription factor family (there are 29 genes in humans), but unlike most of its members (with the exception of SAP1 and SAP2) it has positioned the DNA binding domain N-terminal. This ETS domain is tightly conserved across the family; within the mammalian Elk1 the conservation approaches 100% (Figure 5A). When one extends this alignment across the ETS domains of 12 different proteins (positioned both N- and C-terminally) one remarkable feature emerges, the conservation of the methionine 55 that corresponds to the AUG^sElk1^ codon (Figure 5A). Furthermore, structural data on the Elk1/SAP1 ETS domain has revealed that this Met^55^ makes conserved backbone and base contacts with the dsDNA of the SRE [10]. This role in protein function presumably exerts selective pressure to retain the methionine in the face of a translational control mechanism that could conceivably render it accessible as a start site due to delayed reinitiation. We directly examined the role of the Met^55^ on Elk1-DNA binding using ChIP analysis targeting the c-fos promoter. Since structural data placed Met^55^ close to the dsDNA phosphodiester backbone a Met→Glu mutation was generated in the human Elk1^HA^ cDNA clone (Elk1^HA^ M55E). We targeted a non-conservative change because the ETS domain makes numerous contacts with dsDNA suggesting that the impact of any single mutation on binding could be quite small. As a negative control we used cells transiently over-expressing sElk1^HA^. The 54 aa N-terminal deletion used to generate the human sElk1^HA^ clone removes most of the DNA binding domain (Figure 5A), and this has already been reported to compromise its ability to activate SRE genes [6]. The ChIP assay was performed twice and analysed by qPCR. The ΔCt values (relative to input) were then normalised relative to Elk1^HA^ which was given a value of 1. The analysis confirmed a significant loss in c-fos promoter binding with the sElk1^HA^ (Figure 5B/C). More importantly, the M55E mutation reduced the ChIP signal to ∼60% of the Elk1^HA^ WT value (Figure 5B/C), validating the structural study and supporting a role for Met^55^ in promoter binding.

## Discussion

The mammalian 5′UTR contains a mosaic of regulatory elements that impact on the translational readout. It is becoming increasingly evident that one of these key elements is the uORF. Bioinformatic studies predict that up to 50% of mRNAs have at least one uORF, and these are particularly prevalent in gene transcripts encoding transcription factors and proto-oncogenes [15]. Therefore, it is not surprising to find uORFs in the *ELK1* 5′UTR considering that the major protein product, a transcription factor, plays a central role in the regulation of the immediate early gene response, and hence, cell proliferation. The 5′UTR can also be a dynamic element with heterogeneity arising due to a combination of alternative promoter usage and alternative splicing. As such, these serve to couple events in the nucleus to the translational readout in the cytoplasm. The relative abundance of these 5′ variants can also be cell type specific suggesting that they may impact on cellular physiology presumably by modulating the translational output.

Our studies on the protein readout from the *ELK1* gene were initially motivated by reports identifying a novel N-terminally truncated sElk1 protein in rat neuronal tissue and the observation of alternative splicing within the 5′UTR of at least the human gene [6], [7]. It seemed intriguing to ask if these events were in some way coupled. Our results have demonstrated that these alternative splicing events are conserved in rat, although the nature of the exonII has changed, but may not be occurring in mouse. However, sensitive reporter assays have consistently failed to demonstrate significant de-novo initiation events at the AUG^sElk1^ independent of the species origin, cellular background and TC levels. Furthermore, the results outlined in this communication point to the “sElk1-like” band observed on immunoblots performed with the Ab^SC^ as being non-specific. In fact it would seem that the mammalian *ELK1* gene has put in place an elaborate mechanism to ensure limited or no 40 S access to the internal AUG^sElk1^ codon [6]. Hence, the judicious spacing of multiple out-of-frame iAUGs upstream which ensure efficient “mopping-up” of 40 S subunits emanating from the uORF2 over a broad physiological range of TC levels [8]. Our studies on the mammalian gene led us to propose that the function of the conserved small uORF2 is principally to regulate the Elk1 protein by coupling its expression to intracellular TC levels [8].

The tight conservation of the methionine 55, which corresponds to the AUG^sElk1^ codon, across all the ETS domains whether positioned N- or C-terminally on the polypeptide, suggested a link to protein function. This interpretation is supported by the Elk1-SRE crystal structure [10] and has been further confirmed by our ChIP analysis on the c-fos promoter (Figure 5). In the context of *ELK1*, this functional role presumably exerts selective pressure to retain the methionine in the face of a translational control mechanism (delayed reinitiation downstream of uORF2) that could conceivably render it accessible as a start site. The translational expression of an N-terminally truncated form of Elk1 lacking the ETS domain, namely sElk1, would probably not be without consequence as it has already been reported that the over-expression of a cDNA clone expressing a sElk1 protein interferes with Elk1 function in the nucleus. In conclusion, our results suggest that the conservation of the AUG codon associated with sElk1 expression is coupled mainly to ETS domain function. We cannot exclude that in certain cellular settings delayed reinitiation events may occur at the AUG^sElk1^, possibly mediated by the intervention of as yet unknown trans-acting factors. Nonetheless, these studies highlight the complexities of dissociating the two possible functions of an AUG codon, namely, translation initiation and protein function, particularly in the light of the accumulating data demonstrating that mammalian initiation complexes can access sequences distal from the 5′ cap via a combination of leaky scanning and delayed reinitiation [16], [17].

How do we reconcile our own observations with those previously reported for sElk1 expression? Part of this is due to the non-specificity of the Ab^SC^ antibody and the unfortunate co-migration of the background band at the position of sElk1 (Figure 1). A second reason arises from the use of cDNA clones in which a large part of the 5′UTR has been removed. In the earlier article, the authors deleted exonI for their studies [6]. This removed both the stable RNA structural elements plus the uAUG1 and as a consequence undoubtedly improved overall expression levels, mimicking the phenotype that we observed with the ΔKpn construct (see Figure 4C). However, as with ΔKpn they allowed initiation events to occur at the AUG^sELK1^ (see Figure 4C) [8]. Likewise, when they sought to tag the protein they placed the HA and GFP tags N-terminally, fusing them directly to the Elk1 ORF. Once again the 5′UTR was removed. A similar error was made in another study that examined the potential impact of AKT activation on Elk1 protein translation, once again the authentic 5′UTR was replaced with that derived from the N-terminally fused reporter [11]. Considering the clear impact of the 5′UTR on the translational readout from the *ELK1* mRNA, the conclusions from these types of studies must be treated with caution. Furthermore, the experiments describing a function for the sElk1 protein (differentiation, neurite potentiation) employed transient expression assays using cDNA clones specifically designed to over-express it. A number of reviews have subsequently pointed to a specific role for this sElk1 protein in the CNS although we would now postulate that the transiently expressed N-terminal deletion mutant rather antagonises the normal function of Elk1 in these cells. Indeed, studies with the SRF transcription factor have demonstrated that transiently over-expressed constructs lacking the DBD retain an ability to interfere with gene expression via transcriptional squelching [18].

## Supporting Information

Figure S1 The immunoblots depicted in Figure 1E (panel A) and Figure 4A (panel B) were quantitated using the Quantity One software package (Bio-Rad). The values (the average of each duplicate) for the major protein products were normalised to the t0 value which was set at 100. Protein half-life values were extracted from these curves.
(TIF)
