# Supplementary figures and images for: An AUG Codon Conserved for Protein Function Rather than Translational Initiation: The Story of the Protein sElk1
Source: PLoS One. 2014 Jul 18;9(7):e102890. doi: 10.1371/journal.pone.0102890 (PMC4103894; doi:10.1371/journal.pone.0102890)

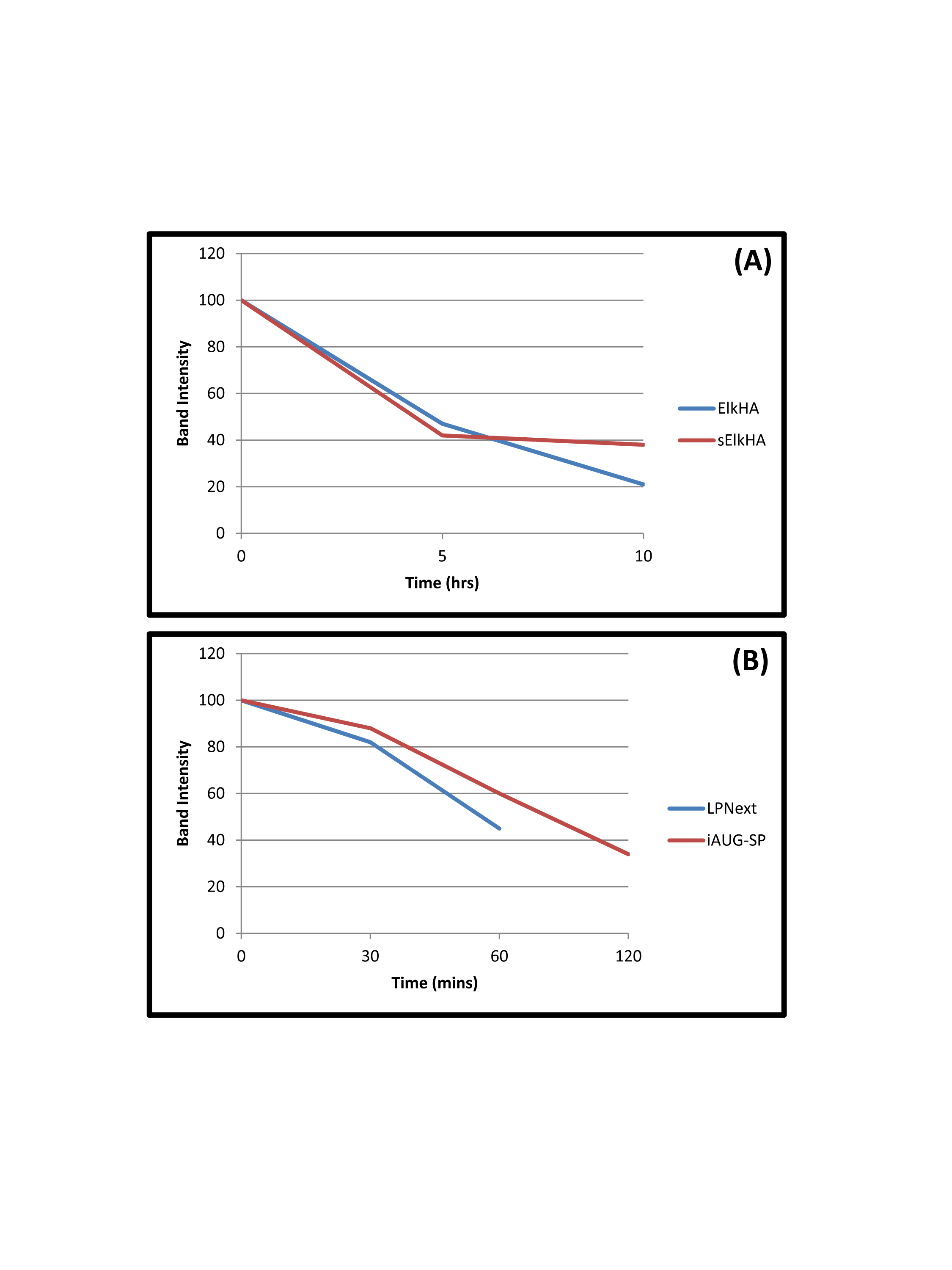

Supplement: Figure S1 — The immunoblots depicted in Figure 1E (panel A) and Figure 4A (panel B) were quantitated using the Quantity One software package (Bio-Rad). The values (the average of each duplicate) for the major protein products were normalised to the t0 value which was set at 100. Protein half-life values were extracted from these curves. (TIF) [file pone.0102890.s001.tif]
